# Elaboration and implementation of a protocol for the Golden Hour of premature newborns using an Implementation Science lens*

**DOI:** 10.1590/1518-8345.6627.3957

**Published:** 2023-07-21

**Authors:** Elizangela Sant’Anna da Silva, Cândida Caniçali Primo, Sarah Gimbel, Márcia Valéria de Souza Almeida, Norma Suely Oliveira, Eliane de Fátima Almeida Lima

**Affiliations:** 1 Universidade Federal do Espírito Santo, Vitória, ES, Brasil.; 2 University of Washington, Child, Family and Population Health Nursing, Seattle, Washington, United States of America.; 3 Universidade Federal do Espírito Santo, Departamento de Pediatria, Vitória, ES, Brasil.

**Keywords:** Preterm Infant, Neonatology, Neonatal Nursing, Clinical Protocols, Implementation Science, Quality Management, Recién Nacido Prematuro, Neonatología, Enfermería Neonatal, Protocolos Clínicos, Ciencia de la Implementación, Gestión de la Calidad, Recém-Nascido Prematuro, Neonatologia, Enfermagem Neonatal, Protocolos Clínicos, Ciência da Implementação, Gestão da Qualidade

## Abstract

**Objective:**

describe the process of designing and implementing a care protocol for the first hour of life of premature newborns.

**Method:**

a participatory research study using an implementation science framework, the Consolidated Framework for Implementation Research (CFIR) was employed to determine drivers and facilitators of implementation success of the Golden Hour protocol for newborns at a large university hospital in southeastern Brazil. A multi-professional team, including first line providers and managers participated in six stages of quality improvement: situational diagnosis; protocol elaboration; training protocol implementation; barrier and facilitator assessment; and protocol monitoring and review. Qualitative and monitoring data collected across these six stages were analyzed using descriptive statistics and content analysis.

**Results:**

the institution’s Golden Hour protocol was organized by the multi-professional team based on a collective and dialogical approach. The protocol prioritized the infant’s cardiopulmonary stability, as well as prevention of hypothermia, hypoglycemia and infection. After four months of implementation, the care team was evaluated the protocol as a good quality intervention, necessary for the service, low-cost and not very complex. One suggested improvement recommended was to carry out refresher training to address staff turnover.

**Conclusion:**

implementation of the Golden Hour protocol introduced an appropriate and feasible neonatal care quality improvement process, which requires periodic refresher training to ensure greater adherence and better neonatal results.

## Abstract (es)

**Objetivo:**

describir el proceso de diseño e implementación de un protocolo de atención para la primera hora de vida del recién nacido prematuro.

**Método:**

investigación participativa, que utilizó el marco de la ciencia de la implementación y los dominios del *Consolidated Framework for Implementation Research*. Estudio realizado en un hospital escuela del sureste de Brasil, con la participación del equipo multidisciplinario y de los gestores. El estudio se organizó en seis etapas, mediante del ciclo de mejora continua (*Plan*, *Do*, *Check*, *Act*): diagnóstico situacional; elaboración del protocolo; capacitaciones; implementación del protocolo; relevamiento de barreras y facilitadores; seguimiento y revisión del protocolo. Los datos fueron analizados mediante estadística descriptiva y análisis de contenido.

**Resultados:**

el primer protocolo de la Hora Dorada de la institución fue organizado por el equipo multidisciplinario a partir de un enfoque colectivo y dialógico. El protocolo priorizó la estabilidad cardiorrespiratoria, la prevención de hipotermia, hipoglucemia e infección. Después de cuatro meses de capacitación e implementación, el protocolo fue evaluado como una intervención de calidad, necesaria para el servicio, de bajo costo y de poca complejidad. La principal sugerencia de mejora fue realizar actividades educativas frecuentes.

**Conclusión:**

la implementación generó cambios e inició un proceso de mejora de la calidad de la atención neonatal, es necesario que la capacitación sea continua para lograr mayor adherencia y mejores resultados.

## Abstract (pt)

**Objetivo:**

descrever o processo de elaboração e implementação de protocolo assistencial para a primeira hora de vida do recém-nascido prematuro.

**Método:**

pesquisa participativa, que utilizou referencial da ciência da implementação e os domínios do *Consolidated Framework for Implementation Research*. Estudo realizado em hospital universitário no sudeste do Brasil, com participação da equipe multiprofissional e gestores. O estudo foi organizado em seis etapas, por meio do ciclo de melhoria contínua (*Plan*, *Do*, *Check*, *Act)*: diagnóstico situacional; elaboração do protocolo; treinamentos; implementação do protocolo; levantamento de barreiras e facilitadores; monitoramento e revisão do protocolo. Os dados foram analisados por estatística descritiva e análise de conteúdo.

**Resultados:**

o primeiro protocolo Hora Ouro da instituição foi organizado pela equipe multiprofissional a partir de uma abordagem coletiva e dialógica. O protocolo priorizou a estabilidade cardiorrespiratória, prevenção de hipotermia, de hipoglicemia e de infecção. Após treinamento e implementação por quatro meses, o protocolo foi avaliado como uma intervenção de qualidade, necessária ao serviço, de baixo custo e pouco complexa. A principal sugestão de melhoria foi realizar ações educativas frequentes.

**Conclusão:**

a implementação provocou mudanças e iniciou um processo de melhoria da qualidade da assistência neonatal, sendo necessária a manutenção dos treinamentos para maior adesão e melhores resultados.

**Highlights:**

**(1)** Employing implementation science study carried out in a university hospital.

**(2)** Organization, implementation and evaluation of the multi-professional care protocol.

**(3)** Collective construction, considering the best available scientific evidence.

**(4)** Transfer of knowledge about neonatal care quality and safety.

**(5)** Translation and application of the best scientific evidence in the work process.

## Introduction

Premature birth is an important risk factor for newborn (NB) morbidity and mortality. The neonatal period presents substantial risk of developing complications in the neonatal period, mainly for extremely premature or extremely low-birth weight infants. Such complications are correlated with longer hospitalization times and unfavorable outcomes, such as sequelae or death^(^ 1 ^-^ 2 ^)^.

The occurrence of increasingly premature births and the reduction in the viability limits are challenges for professionals who assist NBs. The first 60 minutes of a premature NB’s life, referred to as the Golden Hour, are essential for maintaining NB life. The Golden Hour strategy focuses on specific goals, developed in Neonatology^(^ 2 ^-^ 3 ^)^. The Golden Hour strategy proposes a more efficient service based on evidence, structuring multidisciplinary teamwork and effective communication; reinforcing integrated work through standardization of care and the use of clinical protocols^(^ 4 ^-^ 5 ^)^. During the Golden Hour, care team actions are carried out to effectively stabilize the health of premature newborns, via preparation for delivery, timely umbilical cord clamping, maintenance of normothermia, monitoring and provision of adequate respiratory support, transfer to the neonatal unit, vascular access and prevention of hypoglycemia, among other clinical care measures^(^ 3 ^-^ 5 ^)^.

A number of studies have already shown the positive impact of implementing Golden Hour protocols in the prevention and reduction of hypothermia and hypoglycemia rates in the care of preterm newborns (PTNBs) through standardization of care^(^ 1 ^,^ 6 ^-^ 7 ^)^. Offering assistance informed by scientific evidence, the Golden Hour protocols contribute to the systematic use of effective interventions, standardization of clinical practices across providers and settings and rational organization of services, as well as contribute to professionals’ ethical awareness^(^ 1 ^,^ 3 ^,^ 8 ^-^ 9 ^)^.

Quality improvement interventions can be driven by implementation research studies, which adopt and integrate evidence-based practices, and study the main drivers of adoption, implementation and sustainability of these practices, with the aim of improving patient and population health outcomes^(^ 3 ^,^ 10 ^-^ 11 ^)^.

The effective integration of evidence-based interventions into neonatal care, including adoption of advanced technologies have contributed to a significant reduction in neonatal morbidity and mortality rates globally^(^ 1 ^-^ 2 ^)^. Implementation settings which focus on continual improvements in quality have the potential for continually better survival outcomes in PTNBs^(^ 3 ^-^ 7 ^,^ 9 ^-^ 11 ^)^.

Understanding the importance of these findings, and considering that the study institution did not have a defined and implemented Golden Hour protocol for PTNBs, the elaboration and implementation of a protocol duly adapted to the local context was deemed important to favor organization, integration and standardization of care for premature infants in their first hour of life, contributing to patient safety and favoring the adoption, dissemination and maintenance of the recommended care measures. Thus, this study aimed to describe the elaboration and implementation process of a care protocol for the first hour of life of premature newborns.

## Method

### Type of study

A participatory research study was conducted, using the Consolidated Framework for Implementation Research (CFIR) as a lens to guide the design and implementation of the multi-professional care protocol for the Golden Hour. The CFIR 1.0 domains and constructs are available at http://www.cfirguide.org/constructs.html. The following CFIR domains were used: characteristics of the intervention, characteristics of the individuals and the organization, and the implementation process.

### Study locus

The study was carried out in a neonatal unit of a university hospital belonging to the hospital network of the Brazilian Company of Hospital Services (*Empresa Brasileira de Serviços Hospitalares*, EBSERH), in the city of Vitória, state of Espírito Santo, Brazil.

### Participants

The study participants included members of the multi-professional team specifically physicians, nurses, nursing technicians and physiotherapists, responsible for assisting PTNBs. The professionals who were away from care duties for any reason during the study period were excluded.

### Data collection

The study was carried out in six stages, organized within the continuous improvement cycle (Plan, Do, Check, Act), also known as PDCA cycle: situational diagnosis; elaboration of the protocol; training sessions; implementation of the protocol; survey of barriers and facilitators in the implementation process; and monitoring and review of the protocol.

In the first stage, an initial situational diagnosis was carried out using data collected from medical records about the first hour of life of PTNBs born less than 34 weeks of gestation at the study hospital and admitted to the Neonatal Unit during the second semester of 2019. A data collection instrument was prepared and used by the researcher, with data based on national and international recommendations and separated into the following groups: general data; preparation for assistance; care immediately after delivery; transport; and admission to the neonatal unit. The data were collected by the researcher herself from March to April 2020.

In the second stage, a working group was assembled to operationalize the protocol and plan implementation activities. During this stage, the professionals invited were those working in the unit with more than one year of experience in the neonatal unit. This group those in leadership positions (head of the unit and technical manager), physician and professionals from the nursing, medical and physiotherapy teams, working day and/or night shifts were contacted, as researchers felt a broader pool would enhance the comprehensiveness and generalizability of the findings. The working group consisted of 12 health professionals, 100% female; mean age of 43 years old, mean of 16 years of experience in Neonatology; seven participants with specialization as maximum degree, three with a master’s degree, one with a PhD and one participant with intermediate level. Only one participant had not attended any neonatal resuscitation course.

Due to the Coronavirus Disease 2019 (COVID-19) pandemic, data was collected virtually – via three virtual group meetings were held to discuss the protocol, as well as two individual assessments, by email, between June 2020 and May 2021. The *Teams* platform was used for the virtual meetings, which were recorded.

The study objectives were presented in the first meeting, as well as data on the situational diagnosis of the service and a proposal for a pilot protocol based on official documents from the Ministry of Health: Ordinance No. 371 of May 7^th^, 2014^(^ 12 ^)^; Ordinance No. 930 of May 10^th^, 2012^(^ 13 ^)^; and Ordinance No. 2,068 of October 21^st^, 2016^(^ 14 ^)^; recommendations for Resuscitation of Premature Infants < 34 weeks in the delivery room: 2016 Guidelines of the Brazilian Society of Pediatrics^(^ 15 ^)^ and Transportation of high-risk newborns: 2017 Guidelines of the Brazilian Society of Pediatrics^(^ 16 ^)^; guidelines contained in the 2019-2021 Neonatal Guidelines from the National Health Service (NHS)^(^ 17 ^)^ and adapted to the local reality; and in the Santa Joana Group material called Assistance to preterm NBs in the neonatal Intensive Care Unit (ICU): 2019 manual of practical courses of action^(^ 18 ^)^.

The protocol structure followed the standardization of the quality sector of the hospital institution under study. The group meetings lasted from one to two hours. After the second and third meetings, adjustments were made to the protocol content and emailed to the participants for an individual evaluation. At the end of this evaluation period, the content was updated with the suggestions received and emailed for a final assessment, in which the participants indicated whether they fully/partially agreed or if adjustments were required, and cited the suggestions; or even if they fully disagreed. After feedback, the protocol was finalized and forwarded to the hospital’s quality sector, which made the protocol available on the institution’s Intranet.

In the third stage, the training sessions was carried out along with the unit’s permanent education program. The training sessions were publicized electronically to the staff of the neonatal, maternal-child and surgery units, and took place from July to August 2021. They were carried out by the researcher and recorded using the Teams platform.

The protocol was presented during the training, highlighting the objectives and changes in the care provided, the flowchart, the monitoring instrument and, at the end, a space was opened for discussion and suggestions.

At the end of the training, an evaluation of the professionals’ motivation level (high, average, low) before and after the training by the institution was carried out. Other aspects addressed in the evaluation were content adequacy and its applicability to practice and the extent to which new knowledge was transferred to learners; the instructor’s performance, his/her knowledge, didactics and communication; infrastructure and logistics, facilities and devices and hour load; the participants’ performance; the strengths and weaknesses of the activity; and any additional suggestions or comments.

In the week before implementation, an in-service orientation was carried out by the researcher with small groups or individually with team members, to clarify doubts about the main changes in care, in the patient flowchart and in the monitoring instrument used in the pre-delivery units, the Neonatal Intensive Care Unit (NICU), the obstetric center and the surgical center.

In the fourth stage, the time to start using the pilot protocol was established together with the managers and teams. The implementation took place over four months, from September 1^st^ to December 31^st^, 2021. During this period, the researcher provided ongoing support to the team to clarify any doubts if they arose.

In the fifth stage, after using the pilot protocol, the barriers and facilitators in the implementation process were surveyed through a semi-structured questionnaire based on three CFIR domains: characteristics of the intervention, characteristics of the individuals and of the organization, and the implementation process. The semi-structured questionnaire used a 3 point Likert scale: I agree, I partially agree and I disagree. Physicians, nurses, nursing technicians and physiotherapists from the neonatal unit completed questionnaires. The answers to the questionnaire were organized in three sections: facilitators and barriers to the protocol implementation process, suggestions to improve the protocol; and implementation process.

In the sixth stage, which started in November 2021 and ended in February 2022, monitoring and review of the protocol were carried out. Using the same instrument as in the first stage, the researcher collected data from medical records about the first hour of life of PTNBs with less than 34 weeks, born at the study hospital and admitted to the Neonatal Unit during the protocol implementation period.

With these data, a monitoring diagnosis was made, verifying execution of the actions established in the protocol. After analyzing the data, the working group met in person, discussed the results obtained and reviewed the protocol, making necessary changes and inclusions. A voice recorder was used for this meeting.

To enhance use of the protocol, two warning signs were prepared for the team and an illustrative flowchart, in banner format was printed, to be displayed in the neonatal unit.

### Data analysis

The quantitative data were analyzed using descriptive statistics. As for the qualitative data, the recordings of the focus group discussions were transcribed and categorical content analysis was used to organize them into three phases: 1) pre-analysis; 2) exploration of the material; and 3) treatment of the results, inference and interpretation^(^ 19 ^)^.

The working group participants were identified as participant P, in this sequence: P1, P2, P3... The participants of the training sessions were identified as participant T, in this sequence: T1, T2, T3... The participants who answered the questionnaire of barriers and facilitators were identified as participant Q, in this sequence: Q1, Q2, Q3... The (...) code means that part of the statement was omitted.

### Ethical aspects

The project was approved by the Research Ethics Committee under number 1,794,528. The participants were informed about the study and signed the Free and Informed Consent Form after reading it. They were also informed about their right to refuse to participate or to refuse to answer any questions, interrupt the interview or withdraw from the study, at any moment, without providing any information or affecting their service.

## Results

In the first stage, for the initial situational diagnosis, 32 medical records of preterm newborns with gestational age (GA) < 34 weeks were evaluated. Regarding the care provided, it was possible to observe that 50% of the PTNBs required resuscitation; 65.6 % were admitted within the first 30 minutes of life; 62.5% had hypothermia on admission; 56.2% of the records had capillary blood glucose after one hour of life; and 73.3% of intravenous hydrations were checked after one hour of life.

Information such as heat source, use of plastic bag and double cap and complete record of vital signs were not found in more than 50% of the medical charts. In the second stage, based on this situational diagnosis, the working group identified the main weaknesses for organizing the first institutional protocol aimed at assisting PTNBs with less than 34 weeks.

The protocol for the first hour of life for PTNBs with less than 34 weeks contains the duties, competencies and responsibilities of each professional category involved in the care during the period that precedes delivery, the birth moment and the PTNB admission to the neonatal unit. It also addresses the necessary actions for cardiopulmonary stabilization, as well as for the prevention of hypothermia, hypoglycemia and infection. The protocol includes a flowchart of the assistance to be provided to PTNBs in the Golden Hour, as well as the admission bundle for monitoring actions.

During the meetings, the working group pointed out the importance of elaborating a protocol adequate to the local reality and their concern with the scope and inclusion and exclusion criteria for using the protocol.

*Adjust this protocol well (...) so that it becomes useful, clear, and that people can really take from it what’s necessary to improve the assistance provided* (P5).

*(...) wouldn’t the surgical center and pre-delivery have to be included in the scope? (...)” As the patient will be treated both there and here (...)* (P7).

*The protocol for the first hour of life of premature infants is so important that I believe that, even if the baby is not born in our maternity ward, it deserves to be subjected to all the measures that the protocol offers (...)* (P9).

In duties, competencies and responsibilities, it was requested to separate the actions in moments: delivery, transferand admission, with a description for all those involved.

*(...) to simplify (...) separate these many duties into the delivery room, transfer and admission* (P5).

*(...) put the specific duties of each person (...). If you’ve already determined (...) perhaps we can do the assistance that needs to be done in one hour* (P7).

Regarding the therapeutic plan, the group defined the value for ambient temperature, use of heated field, thermal stabilization for carrying out the transfer and verification of maternal temperature in the delivery room. Along with that, the need for equipment for adequate oxygen supply and risk classification for transfers was pointed out.

*I think that it should have a single temperature. Even more if it’s below 1 kg* (P1).

*And the heated field is not part of our routine, we should also change it (...)* (P7).

*The ideal would be to stabilize, improve the temperature, to transfer later* (P2).

*We started measuring the mother’s temperature. Because, sometimes, the mother is hypothermic, and the baby is already born losing (...) having the compressed air bullet with the Blender working. This is more important than anything else* (P11).

*(...) it could be a step forward for us to establish this routine (...) to classify the newborn’s risk (...)* (P9).

Actions related to venous access and radiography were signaled.

*(...) define which access will be based on weight or gestational age?* (P9).

*If it is to catheterize below 1.5 kg, it should be catheterization first* (P11).

*The x-ray is a bit complicated, because sometimes (...) it takes a long time* (P8).

The bundle designed for monitoring the activities also underwent minor adjustments in its content.

After its elaboration, the protocol was evaluated by the working group participants. Seven participants stated that they agreed with the material and some suggestions were made and accepted, three subjects fully agreed with the material, and two participants did not issue any opinion.

In the third stage, with the protocol corrected and approved by the working group, the online training sessions were carried out. 59 professionals participated in the training: 20 nurses, 18 nursing technicians, 10 physiotherapists, six physicians, two resident physicians and two speech therapists (all working in the neonatal unit), and a nurse from the Nursing Division. The reaction assessment was answered by 42 participants, and the high motivation level rose from 59.5% before the training to 81% after the sessions.

The importance of the protocol and training was highlighted in the participants’ testimonies.

*(...) we do many things, but now, with knowledge, we’re even thirstier to do. We can start right?*(T7)

*Most of the time we do the protocol, but we don’t follow this training, you know, and I think that makes all the difference* (T9).

*(...) it’ll be a great benefit for us to improve what we already do* (T14).

A total of 87 professionals participated in the in-service guidance to implement the protocol: 65 professionals from the neonatal unit, five from the pre-delivery and obstetric center, and 17 from the surgical center. However, the surgical center professionals who work in the night shift were not approached.

In the fourth stage, to start using the protocol, an adjustment was made to the Perinatal Clinical History form, including data on maternal temperature at the time of delivery, NB temperature and risk score for transfer. In each transfer incubator, the table for calculating the Morbidity Risk Score during Intra-Hospital Transfer (ERTIH-Neo) was fixed and made available in the unit printed with the admission bundle.

In the fifth stage, after four months of having implemented the protocol, a semi-structured questionnaire was applied, organized under the CFIR domains, which was answered by 44 professionals from the neonatal unit, including nurses, physicians, physiotherapists and nursing technicians. The results are shown in Table1.

**Table 1 -:** Description of the characteristics of the intervention, the individuals and the implementation process based on the CFIR^*^ domains, by nurses, physicians, physiotherapists and nursing technicians from the neonatal unit (n^†^=44). Vitória, ES, Brazil, 2022

CFIR* domains	I agree		I partially agree		I disagree		Did not answer	
	N^†^	%^‡^	N^†^	%^‡^	N^†^	%^‡^	N^†^	%^‡^
**Characteristics of the intervention**								
Is the protocol a need for the service/institution?	41	93.18	3	6.81	0	0	0	0
Is the protocol adapted to meet the local reality?	29	65.90	15	34.09	0	0	0	0
Is the protocol a good quality intervention for the care provided at the unit?	44	100	0	0	0	0	0	0
Is the protocol too complex or has many steps and stages which make it difficult to use?	8	18.18	5	11.36	29	65.90	2	4.54
Did the protocol require many investments to be implemented?	4	9.09	6	13.63	29	65.90	5	11.36
**Characteristics of the individuals**								
Do the neonatal unit professionals support use of the protocol?	35	79.54	9	20.45	0	0	0	0
**Implementation process**								
Can knowing the data on use of the protocol favor maintenance of its use?	44	100	0	0	0	0	0	0

^*^CFIR = Consolidated Framework for Implementation Research; ^†^N = Number; ^‡^% = Percentage

When evaluating the protocol, the participants who partially agreed that it was a need for the service/institution and adapted to meet the local reality mentioned the need to expand its use to other services, the benefit for the PTNB’s family and issues related to human resources, to the physical structure and to the protocol attributions.

In the complexity item, some participants agreed that the protocol is very complex but, even so, they considered the protocol necessary; others linked complexity of the protocol to the procedures and performance of the team. Regarding the investments, the participants highlighted the professionals’ educational investment; five participants did not answer and, of these, some stated not having sufficient knowledge to answer the question.

When evaluating the characteristics of the individuals, 79.5% of the participants answered that the neonatal unit professionals supported use of the protocol. Those who answered that they partially agreed addressed the professionals’ behavior and lack of knowledge related to the protocol.

Through the semi-structured questionnaire, the participants reported possible facilitators and barriers in the protocol implementation process. Figure1 presents the facilitators and barriers from the professionals’ perspective.

**Figure 1 -:** Description of the facilitators and barriers for the protocol implementation process. Vitória, ES, Brazil, 2022

Facilitators	N*	Barriers	N*
Training/Instruction	17	Adherence by the neonatal team and other sectors	26
Commitment by the neonatal multi-professional team	12	Lack of knowledge/training	17
Team organization	5	Physical structure	14
Disclosing of data regarding the protocol	4	Human resources	7
Characteristics of the service	4	Material (missing and in operation)	4
Organization of the materials	3	Communication between sectors	3
Elaboration, publication and dissemination of the protocol	2	Absence of defined duties	2
Use of a checklist	2	Parental knowledge	2
Specialized team	2	Admission complications	1
Institutional structure	2	Lack of organization	1
Use of actions already practiced by the team	1	NB^†^ diversified profile	1
Low turnover of professionals	1		
Nurse’s performance	1		
Institutional demand	1		
Management commitment	1		
Unawareness about the item	1		

^*^N = Number; ^†^NB = Newborn

The suggestions made by the participants for improving the protocol and its implementation were mainly related to continuous training, printed materials in the sectors, periodic review of the protocol, monitoring and actions against non-conformities and the involvement of other sectors.

In the sixth stage, for the monitoring diagnosis after implementation of the protocol, data were collected from 27 medical records and 15 bundles filled-in on admission. In the general data, there was prevalence of cesarean deliveries, during the day shift, and of moderate PTNBs (51.8%), with 8 (29.6%) extreme PTNBs standing out, of which four had GAs below 24 weeks. Regarding care immediately after delivery, the need for resuscitation was observed in 51.8%, with four PTNBs requiring advanced resuscitation.

As for transfers, in 16 (59.2%) medical charts, no records of professionals involved in transfers were found; and, in 11 (40.7%), the transfers were carried out by the Neonatology team, with prevalence of using transfer incubators and respiratory support, this latter employed in 88.8% of the PTNBs.

Regarding admission to the neonatal unit, 51.8% of the PTNBs were admitted with more than 30 minutes of life. However, of the total of 14 PTNBs, six were admitted with less than 40 minutes. Of the PTNBs, 55.5% had hypothermia on admission, 92.5% had respiratory support installed, 62.9% of the capillary blood glucose records were performed in the 1^st^, and 55.5% of the intravenous hydration procedures were checked after one hour of life.

Of the PTNBs admitted in the post-implementation period, 100% used intravenous hydration, including one who received enteral diet in the 1^st^ hour. In relation to this item, it is noted that, among the hydrations administered in the 1^st^ hour of life, only one was through an umbilical catheter, which is a temporary catheter inserted in the delivery room; the other hydrations were applied through peripheral venous accesses. Only one PTNB received a surfactant dose in the 1^st^ hour of life and 59.2% used antibiotics on the first day of life; however, only one PTNB received a dose of antibiotics in the first hour of life and 74% of the medical charts had incomplete records of vital signs at the end of the first hour of life.

In general, during collection of the monitoring data, there was a reduction in the amount of data without records.

**Figure 2 -:**
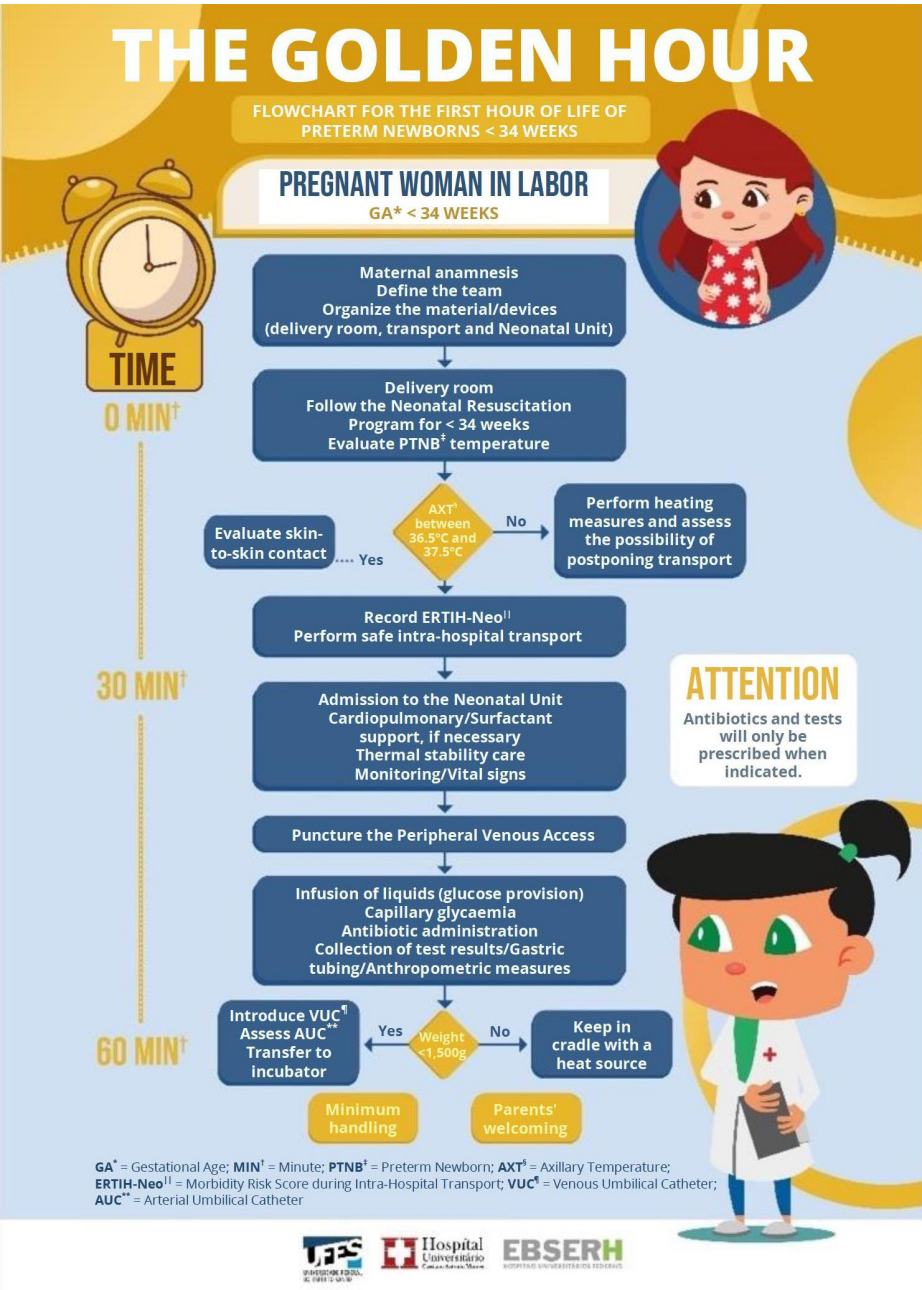
Illustrative flowchart of the Golden Hour protocol. Vitória, ES, Brazil, 2022

^*^IG = Gestational Age; ^†^MIN = Minute; ^‡^RNPT = Preterm Newborn; ^§^TAX = Axillary Temperature; ^||^ERTIH-Neo = Morbidity Risk Score during Intra-Hospital Transfer; ^¶^CUV = Venous Umbilical Catheter;^**^CUA = Umbilical Arterial Catheter

**Figure 3 -:**
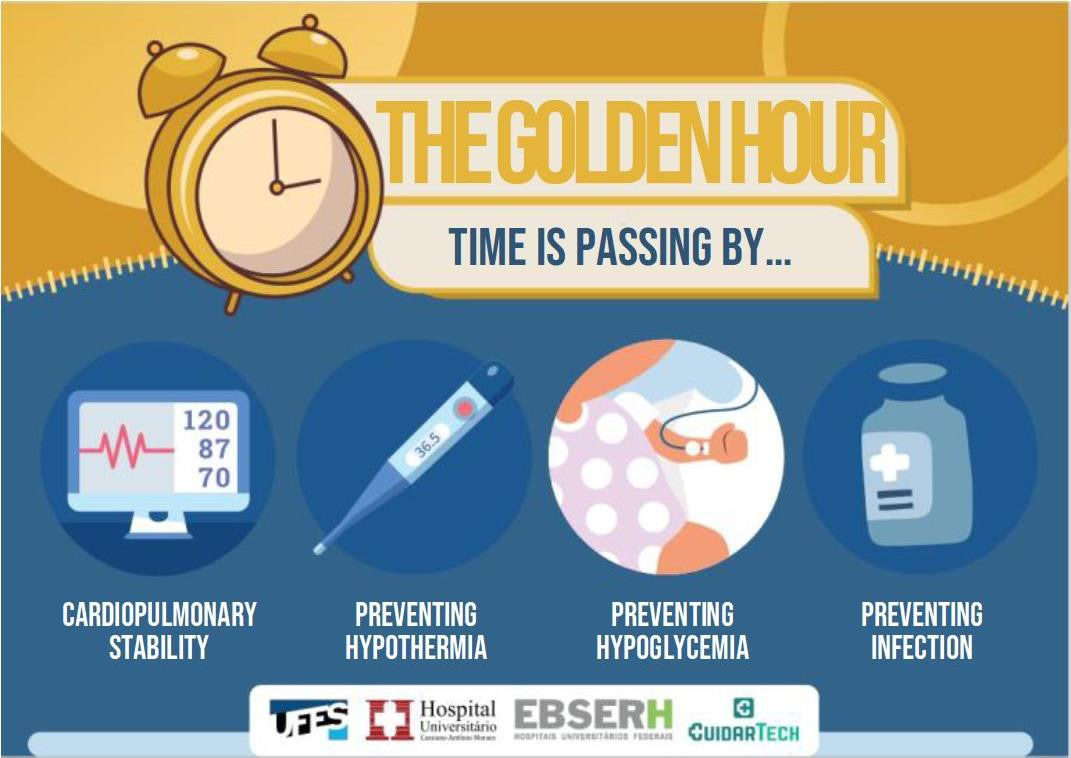
Golden Hour warning sign. Vitória, ES, Brazil, 2022

**Figure 4 -:**
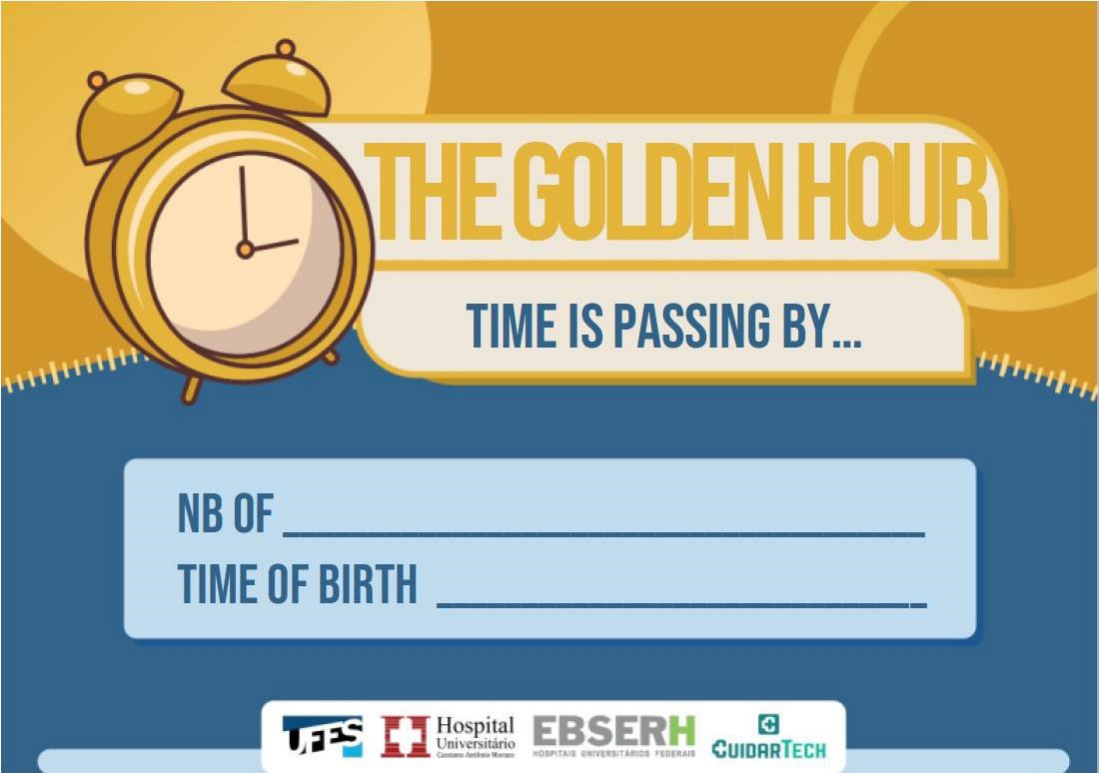
Sign to indicate the time of birth. Vitória, ES, Brazil, 2022

In view of the results, in the sixth stage, the working group reviewed the protocol. The group scored questions in relation to thermal stabilization of the PTNBs, heating of the resuscitation bed, first-choice venous access to optimize glucose solution supply, checking vital signs, administration of antibiotics, and time control during that first hour. Adjustments were also made to the admission bundle. Finally, a proposal for educational signs was presented to the group to alert the team about care during the golden hour. The proposal was accepted by the group, which suggested the words and possible illustrations to be used, which are represented in Figures 2,3 and 4.

## Discussion

In Brazil, more than 308,000 premature births were recorded in 2020; among these, 13,646 corresponded to NBs with gestational ages between 22 and 27 week^(^ 20 ^)^. In this study, the data demonstrate that premature births of extreme PTNBs, specifically those requiring intubation and advanced resuscitation, increases the need for more complex interventions for their clinical stabilization.

International studies highlight the importance of implementing protocols, bundles or checklists as a strategy for improving the care provided to PTNBs^(^ 3 ^-^ 5 ^,^ 9 ^-^ 10 ^)^. Following the international recommendations, the protocol elaborated in this study was organized under four main pillars: cardiopulmonary stabilization, and prevention of hypothermia, hypoglycemia and infection. Actions such as setting the ambient temperature, using a heated field, equipment for fractional oxygen supply and agility in obtaining venous access are important to meet these pillars, as observed in other studies^(^ 3 ^-^ 4 ^,^ 9 ^-^ 10 ^)^.

For the use of these strategies to achieve positive results, it is crucial to have a team duly prepared for this assistance, as PTNBs < 34 weeks may require resuscitation and supplemental oxygen supply^(^ 21 ^)^. In this study, 50% of the PTNBs required resuscitation in the delivery room, at the initial diagnosis.

National and international research studies indicate that postpartum hypothermia poses a risk to the vitality of PTNBs and that it is a predictive factor of morbidity and mortality, regardless of gestational age. Heat loss cannot be overcome by heat production, mainly in premature infants, and the team must carry out interventions for thermal control while caring for the PTNB^(^ 4 ^,^ 6 ^,^ 22 ^-^ 23 ^)^.

At a high-performance NICU, the prevention of hypoglycemia and infection and delayed cord clamping are also important aspects of care to be provided to PTNBs after birth^(^ 3 ^,^ 5 ^,^ 10 ^)^. For the prevention of infections, it is necessary to adopt aseptic measures in PTNB handling, as well as adequate administration of antibiotics^(^ 3 ^)^.

The data after implementation of the protocol revealed advances such as reduction in the hypothermia rate on admission, as well as an increase in capillary blood glucose levels and in the glucose solution supply in the first hour of life. A research study conducted in two Brazilian hospitals verified successful results in preventing hypothermia in very low birth weight preterm infants, using standardization of care in the delivery room and team training^(^ 7 ^)^. International studies report improvements in PTNB glycaemic and temperature control after implementation of the Golden Hour Protocol^(^ 3 ^-^ 6 ^,^ 10 ^,^ 23 ^)^, supporting the results of this study.

Corroborating our findings, two international studies also found a prolonged time to obtain venous access, administration of fluids and antibiotics, routine use of umbilical catheter, and low use of peripheral venous catheter^(^ 3 ^,^ 5 ^)^. Considering the recommendation of peripheral venous access and subsequent umbilical catheterization to prevent hypoglycemia related to the delay in fluid supply^(^ 1 ^,^ 23 ^)^, the team adopted this approach in the protocol.

In general, there was an improvement in the records of the care provided in the first hour of the PTNBs by the professionals after implementing the protocol. The use of protocols based on the best evidence, adapted to the local context and implemented by a trained team with good communication, can reduce variability of the actions, promote safety, improve the birth experience and reduce unnecessary procedures^(^ 3 ^-^ 6 ^,^ 24 ^)^. When evaluating the implementation, training was the main cited facilitator, while team adherence, mainly from other sectors, and lack of knowledge were the most cited barriers. The need for continuous training of the professionals, with the involvement of the multi-professional teams, was also mentioned in other studies as a facilitating component, capable of promoting active participation in processes and avoiding decline of the proposal^(^ 10 ^,^ 23 ^,^ 25 ^)^.

Another relevant factor for maintaining good practices is monitoring intervention implementation. Some successful improvements may be lost over time, resulting in team members returning to the use of old practices. Therefore, incentives are needed to maintain quality and safety, like training and ongoing monitoring^(^ 3 ^,^ 10 ^-^ 11 ^,^ 25 ^)^.

By using implementation science approaches, it is possible to carry out a comprehensive and contextually tailored analysis and adopt effective measures, consistent with the demands of the patient, the team and the service^(^ 11 ^)^. Supported by this concept, this study describes how to translate and apply the most up-to-date scientific evidence into the work process, organizing qualified care, ensuring the professional standardization of actions based on the safety and quality principles, and advancing the translation of knowledge into assistance and management contexts.

Organization of the protocol considered the best available evidence, professional experience and existing resources. However, it did not meet the premise of considering the patients’ preferences, as it was not validated by the users, which constituted a limitation of this study. A further limitation worth noting are related to the conditions imposed by the COVID-19 pandemic and the subsequent restructuring in the study site due to the pandemic, which interfered with the progression of the paper and with adequate adherence by the collaborators to the study.

Other limitations were observed, such as the initial situational diagnosis and monitoring with different quantitative and profiles making it impossible to compare all data before and after the intervention, as well as not training 100% of the professionals of the units involved in the assistance to PTNBs. Given these considerations, new studies are suggested to evaluate use of the protocol and its results in the medium- and long-term, bearing in mind that the current study shows the initial improvements achieved in the short term, with the implementation of the first institutional protocol.

This study contributed to scientific knowledge advancement by describing how to design and implement a Golden Hour protocol for PTNBs, based on scientific evidence, adapted to the local context and with the involvement of the multi-professional team to ensure success of the initiative.

## Conclusion

The institution’s first Golden Hour protocol was organized by the multi-professional team based on a collective and dialogical approach. The protocol prioritized cardiopulmonary stability, as well as prevention of hypothermia, hypoglycemia and infection. The study findings indicate that this collective construction has the potential to meet the demands of PTNBs, the local reality and assist professionals in decision-making.

In addition to the protocol, unpublished educational materials were produced and posted across the institution’s sectors for the health professionals, patients and families with an appealing and creative presentation, transmitting information in a clear and direct way (illustrative flowchart of the “Golden Hour” protocol; “Golden Hour” warning sign and sign indicating the time of birth). These materials configure technologies in the translation of knowledge about patient safety for improving care and management in Neonatal Nursing.

The protocol for assisting PTNBs with less than 34 weeks during the first hour of life has already been implemented and is in use in the study site. Compliance with all actions within the protocol has not yet been measured, but its adoption already represents an improvement in terms of quality, as the unit did not have systematic way to manage PTNB care prior to the study. Thus, with protocol implementation, a process to change professionals’ behavior and knowledge in relation to quality and safety of the PTNB care provided at the Golden Hour was initiated.

Consolidation of the evidence-based care recommended in the protocol requires continuing education of the neonatal team and other sectors involved, in order to assist in adherence and in achieving better results, which may be expanded to include professors and health students in the future. It is also noted that the acquisition of materials favors the achievement of better results.
